# Healthful and Unhealthful Plant-Based Diets and Their Association with Cardiometabolic Targets in Women Diagnosed with Breast Cancer: A Cross-Sectional Analysis of a Lifestyle Trial

**DOI:** 10.3390/nu17233782

**Published:** 2025-12-02

**Authors:** Sara Vitale, Elvira Palumbo, Angela D’Angelo, Matteo Di Maso, Jerry Polesel, Maria Grimaldi, Giuseppe Porciello, Assunta Luongo, Rosa Pica, Anna Crispo, Ilaria Calabrese, Luca Falzone, Michelino De Laurentiis, Vincenzo Di Lauro, Daniela Cianniello, Ernesta Cavalcanti, Anita Minopoli, Marco Cuomo, Renato de Falco, Guglielmo Thomas, Massimiliano D’Aiuto, Massimo Rinaldo, Samuele Massarut, Agostino Steffan, Francesca Catalano, Francesco Ferraù, Rosalba Rossello, Francesco Messina, Vincenzo Montesarchio, David J. A. Jenkins, Gabriele Riccardi, Carlo La Vecchia, Massimo Libra, Egidio Celentano, Livia S. A. Augustin

**Affiliations:** 1 Epidemiology and Biostatistics Unit, Istituto Nazionale Tumori IRCCS “Fondazione G. Pascale”, 80131 Naples, Italy; sara.vitale@istitutotumori.na.it (S.V.); l.augustin@istitutotumori.na.it (L.S.A.A.); 2 Department of Clinical Sciences and Community Health, University of Milan, 20122 Milan, Italy; 3 Unit of Cancer Epidemiology, Centro di Riferimento Oncologico di Aviano (CRO) IRCCS, 33081 Aviano, Italy; 4 Healthcare Direction “A. Cardarelli” Hospital, 80131 Naples, Italy; 5 Department of Biomedical and Biotechnological Sciences, Oncologic, Clinical and General Pathology Section, University of Catania, 95124 Catania, Italy; 6 Division of Breast Medical Oncology, Department of Breast and Thoracic Oncology, Istituto Nazionale Tumori IRCCS “Fondazione G. Pascale”, 80131 Naples, Italy; 7 Laboratory Medicine Unit, Istituto Nazionale Tumori IRCCS “Fondazione G. Pascale”, 80131 Naples, Italy; 8 Clinica Mediterranea, 80122 Naples, Italy; 9 Breast Unit, Ospedale di Boscotrecase, 80042 Boscotrecase, Italy; 10 Breast Surgical Oncology Unit, Centro di Riferimento Oncologico di Aviano (CRO) IRCCS, 33081 Aviano, Italy; 11 Immunopathology and Cancer Biomarkers Unit, Centro di Riferimento Oncologico di Aviano (CRO) IRCCS, 33081 Aviano, Italy; 12 Breast Unit, Cannizzaro Hospital, 95021 Catania, Italy; 13 Breast Unit, Ospedale San Vincenzo, 98039 Taormina, Italy; 14 Breast Unit, Ospedale Evangelico Betania, 80147 Naples, Italy; 15 Division of Medical Oncology, AORN dei Colli-Monaldi Hospital, 80131 Naples, Italy; 16 Department of Nutritional Sciences and Medicine, Temerty, Faculty of Medicine, University of Toronto, Toronto, ON M5S 1A8, Canada; 17 Clinical Nutrition and Risk Factor Modification Centre, St. Michael’s Hospital, Toronto, ON M5B 2T2, Canada; 18 Division of Endocrinology and Metabolism, St. Michael’s Hospital, Toronto, ON M5B 2T2, Canada; 19 Li Ka Shing Knowledge Institute, St. Michael’s Hospital, Toronto, ON M5B 2T2, Canada; 20 Department of Clinical Medicine and Surgery, Federico II University, 80131 Naples, Italy

**Keywords:** breast cancer survivors, healthy plant-based diet, PDI, cardiometabolic risk factors, macronutrients, micronutrients, dietary fiber

## Abstract

Background: Plant-based diets are recommended in guidelines for the prevention of cancer and cardiometabolic diseases, which remain major causes of death in breast cancer survivors (BCS). Since not all plant foods are healthy, we calculated the plant-based dietary index (PDI), healthy (hPDI) and unhealthy (uPDI), and their associations with cardiometabolic targets in BCS. Methods: Baseline dietary and cardiometabolic data were derived from 492 (median age 51, IQR 46–59) female BCS participating in a multicentric lifestyle trial conducted in Italy. Dietary data were collected with 7-day food records. PDI, hPDI, and uPDI were calculated by assigning positive scores to all plant foods, healthy plant foods or less healthy plant foods, respectively, as defined by the literature (scores ranged from 18 to 90). Using logistic or multinomial regression models, we estimated the odds ratios (OR) and the corresponding 95% confidence intervals (CI) between PDIs and cardiometabolic risk factors. Results: The OR of being obese (BMI ≥ 30 Kg/m^2^) was 0.47 (95%CI: 0.29–0.77), 0.37 (95%CI: 0.22–0.61) and 1.38 (95%CI: 0.83–2.28) with higher PDI, hPDI and uPDI, respectively. The OR of having a large waist circumference (≥88 cm) was 0.64 (95%CI: 0.42–1.00) with higher hPDI. The OR for hypercholesterolemia (≥200 mg/dL) was 1.80 (95%CI: 1.16–2.78) with higher uPDI. The ORs of hypertriglyceridemia (≥150 mg/dL) and metabolic syndrome were 0.38 (95%CI: 0.20–0.71) and 0.59 (95%CI: 0.35–0.97), respectively, with higher PDI. No other significant association was observed. Conclusions: Maintaining cardiometabolic risk factors within normal ranges is clinically relevant in BCS, and this may be more likely when a plant-based diet is consumed, especially if low in unhealthy plant foods.

## 1. Introduction

Breast cancer is the most frequently diagnosed female cancer worldwide [1]. The 5-year relative survival in developed countries has reached 92% [2], albeit with an intrinsic higher risk of comorbidities, such as cardiovascular diseases (CVD) and type 2 diabetes (T2D) [3,4]. Low-quality diets represent the second cause of cardiovascular burden, responsible for 44% of all CVD deaths globally [5], while higher diet quality has been advocated by international guidelines for the prevention of CVD and cancer and for reduced mortality [6,7,8,9,10,11].

“Plant-based diet” is a general term for various types of vegetarian dietary patterns that discourage some or all animal foods [12,13]. Evaluating diets only as a dichotomy of vegetarian and omnivorous has limitations [14] since not all plant foods are beneficial to general health, cardiometabolic and breast health [12,15,16]. Studies on healthy subjects reported that higher consumption of whole grains, nuts, fruits and vegetables was associated with lower premature mortality [17,18], while opposite results were found for plant foods, such as refined grains, potatoes, commercial foods and drinks with abundant added sugars and salt [19,20]. Thus, Satija et al. [15] proposed a new approach to the plant-based dietary index (PDI), designed to assess the overall diet while differentiating healthy from less healthy plant foods: healthy plant-based dietary index (hPDI) and unhealthy plant-based dietary index (uPDI). These dietary indices have been investigated for their relationship with risk of CVD, T2D and total mortality in non-oncologic populations [12,15,21,22,23,24]. In these cohort studies, the hard endpoint was inversely associated with hPDI and directly with uPDI. In a cross-sectional study of adults with chronic disease, uPDI was associated with higher fasting glycemia, circulating total and low-density lipoprotein cholesterol levels, while hPDI was significantly inversely associated with total serum cholesterol concentrations [25]. In BCS, the only study investigating hPDI in relation to cardiometabolic conditions (i.e., T2D, hypertension, dyslipidemia) is the Pathways study [26]. However, no study investigated PDI, hPDI and uPDI concomitantly in relation to CVD risk factors and metabolic syndrome in BCS. Therefore, since BCS are at higher risk of cardiometabolic conditions [27,28], we investigated the relationship of PDI and its healthy and less healthy versions with cardiometabolic targets in BCS living in a Mediterranean country.

## 2. Materials and Methods

### 2.1. Overview of the DEDiCa Study

This cross-sectional study, planned according to the STROBE guidelines (Table S1), is part of a multicenter randomized controlled trial of the effect of an intervention, including dietary modification, physical activity and vitamin D supplementation (DEDiCa Study) in breast cancer recurrence [29]. The study was registered with ClinicalTrials.gov (NCT02786875; EudraCT n. 2015-005147-14). The study protocol was approved by the “Agenzia Italiana del Farmaco” (AIFA-Italian Drug Agency), the Ethic Board of the Italian Medicine Agency (AIFA/RCS/P/25054) and of each recruiting hospital (specified at the end of the manuscript in the Institutional Review Board Statement).

Participants (n = 506) were recruited from November 2016 to July 2021 and were followed up in national cancer institutes or oncologic departments in three regions of Italy: Campania (South), Sicily (South) and Friuli Venezia Giulia (North) [29].

Eligible participants were found through surgical lists of participating hospitals, were contacted by phone and were offered to attend group information sessions. Consenting subjects were randomized to one of two lifestyle treatments to be followed for approximately three years. The inclusion criteria were as follows: primary diagnosis of histologically confirmed breast cancer (stages I–III, if stage I, then Ki67 ≥ 30%) within 12 months from diagnosis; age ≥30 and <75 years; ability to comprehend and willingness to sign the consent form, and to adhere to the protocol, including scheduled clinic visits and assigned treatment. The exclusion criteria were as follows: patients with sarcoidosis or other granulomatous diseases or with hypercalcemia (Ca > 11 mg/dL); patients with any previous or current concomitant malignant cancer; pregnant or lactating women; patients with AIDS diagnosis; patients with severe renal insufficiency; patients with kidney stones (nephrocalcinosis or nephrolithiasis); patients participating in other lifestyle clinical trials. More information regarding the study design can be found in the original publication [29].

For this cross-sectional analysis, we included 492 DEDiCa eligible participants who completed the baseline visit.

### 2.2. Dietary Assessment and the Plant-Based Dietary Indices (PDIs)

Dietary data, including beverages, were recorded in grams for 7 days by participants before their study visits and were collected and reviewed by research dietitians at baseline. Data were stored and processed using a professional nutritional analysis software (WinFood©) which utilizes two Italian nutrition databases: “CREA—Alimenti e Nutrizione” (Consiglio per la Ricerca in Agricoltura e l’Analisi dell’Economia Agraria) and “BDA—banca dati di composizione degli alimenti per studi epidemiologici in Italia”. Glycemic index (GI) values were derived from the Italian GI tables [30] and from the “International tables of glycemic index and glycemic load values”, where GI values were determined according to “Food and Agriculture Organization”/”World Health Organization” (FAO/WHO) and ISO (International Organization for Standardization) standards [31,32]. These GI values were preferentially chosen among Italian foods because they were prepared according to the Mediterranean culinary traditions and because they were tested by GI laboratories following the standard methodology. The GI values were inserted in the software WinFood by study dietitians to create a GI folder of commonly consumed carbohydrate foods by study subjects.

Using the dietary data extracted from Winfood and the methodology defined by Satija and colleagues [12], three plant-based diet indices were calculated: an overall PDI, an hPDI and an uPDI. In line with Satija et al., we identified 18 food groups and divided them into healthy plant foods (i.e., whole grains and pasta, vegetables, fruits, legumes, nuts, vegetable oils and tea/coffee), less healthy plant foods (i.e., highly refined grains, fruit juices, sugar-sweetened beverages, potatoes and sweets/desserts) and animal sources of food (i.e., meat and miscellaneous animal-based foods, animal fats, eggs, dairy, fish/seafood). Pasta was included among healthy plant foods for several reasons. It is made of durum wheat semolina by Italian law n. 580 (in 1967 and modified in 2001), a course type of flour, which contains intact cell walls [33], unlike its refined counterpart. According to the Food and Drug Administration definitions, pasta is a “good source” of dietary fiber (10–19% of daily reference value (DRV) of 28 g/day; 100 g dry pasta contains 4 g of dietary fiber or 14% DRV) [34]. Pasta is a staple carbohydrate food of the Italian Mediterranean diet, and it has a low glycemic index, which helps to control glycemia, cholesterolemia, blood pressure and body weight in randomized trials, while in epidemiological observations it is associated with lower risk of cardiometabolic diseases and cancer [35,36]. Overall, durum wheat semolina pasta, within an energy-balanced diet, has been associated with health benefits and lack of negative effects, including excess body weight otherwise observed with foods made of soft wheat refined flours [37].

Each food group was expressed in grams, divided into quintiles of consumption in grams per day and assigned a score from 1 to 5. PDI was calculated by assigning incremental positive scores (i.e., the higher the daily intake, the higher the score) to quintiles of plant foods and reverse scores to animal foods, hPDI by assigning positive scores to healthy plant foods and reverse scores (i.e., the higher the daily intake, the lower the score) to animal and less healthy plant foods, while uPDI by assigning positive scores to less healthy plant foods and reverse scores to animal and healthy plant foods. Scores ranged from 18 to 90, the lowest score indicating lower adherence to PDI, hPDI or uPDI.

Adherence to the Mediterranean diet was estimated with the validated Mediterranean Diet Adherence Screener (MEDAS) questionnaire developed to study the dietary compliance to the Mediterranean diet within the Spanish intervention trial PREDIMED (Prevencion con Dieta Mediterranea) [38]. MEDAS consists of 14 questions on the consumption of typical and non-typical Mediterranean foods throughout the year. The maximum score is 14, indicating the highest adherence to the Mediterranean diet, while 0 indicates the lowest adherence [39].

### 2.3. Cardiometabolic Assessment

Participants’ weight, height and waist circumference were obtained by trained investigators. Height was measured to the nearest 1 cm using a Seca stadiometer, and weight was measured to the nearest 0.5 Kg using a scale (Seca 761). Body mass index (BMI) was calculated as body weight in kilograms divided by the square of the height in meters, and cut-offs were found from the WHO guidelines: normal BMI ≥ 18.5 < 25 kg/m^2^, overweight BMI ≥ 25 kg/m^2^ < 30 kg/m^2^, obesity BMI ≥ 30 kg/m^2^ [40]. Waist circumference was measured in centimeters (cm) at the level of the umbilicus with a non-elastic measuring tape, and the cut-off (<88 cm) was obtained from the metabolic syndrome definition of the National Cholesterol Education Program Adult Treatment Panel III-2001 [41].

Systolic and diastolic blood pressure and heart rate were measured with a digital automatic blood pressure monitor (Nissei, DS-11, CA-MI s.r.l., Pilastro-PR, Italy) after 10 min of rest while sitting and repeated three times, from which the average value was derived. The cut-offs for systolic and diastolic blood pressure were <130 mmHg and <80 mmHg, respectively [42]. Serum values were obtained from centrifugation of venous blood samples collected in Vacutainer tubes without anticoagulant (Becton, Dickinson and Co, Milano, Italy). Total cholesterol, triglycerides, high-density lipoprotein cholesterol (HDL-C), low-density lipoprotein cholesterol (LDL-C) and glucose serum concentrations were determined by spectrophotometric methods on the Cobas C6000 automated analyzer (Roche Diagnostics, S.p.A., Monza-MB, Italy) according to the manufacturer’s instructions. Hemoglobin A1c (HbA1c) value was determined using whole blood collected in EDTA Vacutainer tubes (Vacutainer; Becton, Dickinson and Co, Milano, Italy) by a turbidimetric inhibition latex immunoassay (TINA QUANT Roche Diagnostics) on Cobas C6000 analyzer (Roche Diagnostics, S.p.A., Monza-MB, Italy). The Diagnostic Laboratory Unit of Istituto Nazionale Tumori—IRCCS “Fondazione Giovanni Pascale” in Naples performed all the above-mentioned analyses under internal and external quality control procedures. The cut-off for serum LDL-C (<116 ng/mL) was obtained from the 2019 European Society of Cardiology (ESC) and European Atherosclerosis Society (EAS) Guidelines [42,43]. The cut-off for serum HDL-C (>50 ng/mL) was obtained from the National Cholesterol Education Program (NCEP) [41] expert panel on detection, evaluation, and treatment of high blood cholesterol in adults (Adult Treatment Panel III) [41]. The cut-off for serum triglycerides (<150 mg/dL) was obtained from the ESC guidelines [42].

Metabolic syndrome was defined according to Alberti et al., 2009 [44], which includes five parameters: triglycerides, HDL-C, blood pressure, fasting glucose and obesity. Three or more abnormal findings out of five would qualify a person as having metabolic syndrome.

### 2.4. Statistical Analyses

Categorical variables were summarized as counts and percentages. Adherence to PDI, hPDI and uPDI was categorized as “Low” and “High” according to median values that were 52, 58 and 53, respectively. Difference in socio-demographic and clinical characteristics according to level of adherence to each index was evaluated through the χ^2^ test. Missing values were few and, therefore, were not substituted prior to the analysis (n = 5 for physical activity; n = 10 for blood pressure; n = 1 for LDL- and n = 1 for HDL-cholesterol).

Logistic or multinomial regression models (when appropriate) were used to estimate the odds ratios (OR), with the corresponding 95% confidence intervals (CI), including adjustments for study centre, age, years of education, physical activity, smoking status, energy intake, menopausal status, adjuvant chemotherapy and endocrine therapy. A DAG showing the covariates used to adjust the estimates of BMI related to PDI, hPDI and uPDI is provided in the Supplementary Materials (Figure S1). The same DAG applies to all other outcome variables considered. Estimates of OR and 95% CI were obtained excluding patients treated for systolic and diastolic blood pressure, glycaemia and glycated haemoglobin and cholesterol.

Student’s t statistic was used to test differences in nutrient intakes between higher and lower PDIs median scores.

Statistical significance was claimed for *p* < 0.05. All analyses were performed using the statistical software R version 4.0.5.

## 3. Results

### 3.1. Characteristic of Study Participants

Participants (n = 492) were recruited from three different regions of Italy: 13% from Sicily, 14% from Friuli Venezia Giulia and 73% from Campania (Table 1). Approximately 57% were aged 50 years or older, and 90% were postmenopausal (natural or pharmacological). Approximately half were physically active, 33% graduated from high school or university, 47% reported never being smokers and 21% were current smokers. Approximately 18% were on a chemotherapy regimen, while 47% had just finished before enrollment and 67% had already started endocrine therapy. Overweight and obesity were prevalent in 61% of the participants, and 69% had a high waist circumference of at least 88 cm. Elevated systolic and diastolic blood pressure were present in 27% and 25% of participants, respectively. Elevated fasting serum glucose or elevated HbA1c levels were present in 21% and 12% of participants, respectively. High serum levels of total cholesterol and LDL-C were present in 43% and 58% of participants, respectively, while high serum triglyceride levels were detected in 15% of participants. Overall, 32% of our patients presented with metabolic syndrome (three to five cardiovascular risk factors). The distributions were significantly different among PDI categories for geographical area, education, physical activity, smoking status, energy intake, BMI, systolic blood pressure, triglyceride levels and metabolic syndrome. Among hPDI categories, significant differences were found in geographical area, age, energy intake and BMI, while for uPDI, differences were in geographical area, education, physical activity, energy intake, BMI and total cholesterol.

Table 2 shows the median daily intake in grams/day of the eighteen food groups included in the calculations of the three PDIs. The intakes of vegetables, legumes, fruits, nuts and vegetable oils were below recommendations in at least 50% of participants, and sweets were consumed above recommendations and above the amounts typically seen in the traditional Italian Mediterranean diet [45,46]. Furthermore, among the animal foods, the dairy product intakes were highest, followed by meat and fish/seafood.

Table S2 and Figure S2 show the distribution of macro- and micronutrients according to the median values of the PDIs. Patients with higher median PDI scores (≥ 52), compared to PDI scores lower than the median (<52), had higher adherence to the Mediterranean diet (MEDAS score: 8.4 ± 1.9 vs. 7.5 ± 1.9; *p* < 0.001), higher intakes of total energy (1492 ± 337 vs. 1344 ± 311 Kcal; *p* < 0.001), dietary fiber (13.8 ± 4.0 vs. 11.5 ± 3.7 g/1000 Kcal; *p* < 0.001), and lower intakes of saturated fatty acids (SFA, 10.6 ± 2.8 vs. 11.7 ± 2.5 g/1000 Kcal; *p* < 0.001) and dietary cholesterol (106.1 ± 36.6 vs. 132.1 ± 45.1 mg/1000 Kcal; *p* < 0.001).

Patients with higher hPDI scores than the median (≥58), compared to lower hPDI (<58), had higher adherence to the Mediterranean diet (MEDAS score: 8.6 ± 1.8 vs. 7.3 ± 1.9; *p* < 0.001), higher dietary fiber intake (14.6 ± 4.0 vs. 10.6 ± 2.8 g/1000 Kcal; *p* < 0.001), and lower intakes of total energy (1379 ± 315 vs. 1473 ± 345 Kcal; *p* < 0.002), SFA (10.2 ± 2.6 vs. 12.1 ± 2.6 g/1000 Kcal; *p* < 0.001) and dietary cholesterol (112.2 ± 44.2 vs. 124.4 ± 40.2 mg/1000 Kcal; *p* < 0.001).

Patients with higher uPDI scores than the median (≥ 53), compared to lower uPDI (<53), had lower adherence to the Mediterranean diet (MEDAS score: 7.4 ± 1.9 vs. 8.7 ± 1.8; *p* < 0.001), and lower intakes of total energy (1355 ± 325 vs. 1515 ± 322 Kcal; *p* < 0.001) and dietary fiber (11.4 ± 3.5 vs. 14.5 ± 3.9 g/1000 Kcal; *p* < 0.001), and higher intakes of SFA (11.4 ± 2.8 vs. 10.8 ± 2.6 g/1000 Kcal; *p* < 0.02) and dietary cholesterol (112.5 ± 40.9 vs. 125.3 ± 44 mg/1000 Kcal; *p* < 0.001). Other macronutrients did not show physiologically significant differences and were balanced between high vs. low scores.

Daily average intakes of micronutrients such as potassium, magnesium, calcium, iron, folic acid, alpha-tocopherol and beta-carotene were significantly higher, while sodium was significantly lower with higher PDI and hPDI and with lower uPDI compared to their counterparts. The largest differences in micronutrient consumption were seen with the higher hPDI category, especially for potassium with more than 300 mg/1000 Kcal/day, beta-carotene with more than 400 mg/1000 Kcal/day and approximately 180 mg less sodium/day compared to lower hPDI scores. A higher hPDI showed higher daily intakes per 1000 Kcal of the heart-healthy minerals and antioxidant vitamins than the higher PDI category.

### 3.2. PDIs and Cardiometabolic Targets

**Figure 1:**
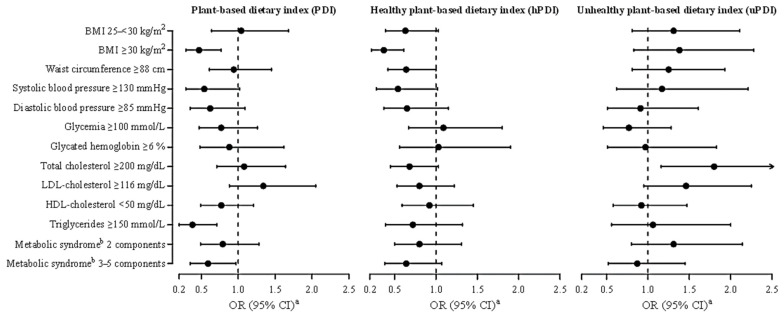
Odds ratios ^a^ (ORs) and corresponding 95% confidence intervals (CIs) for the associations between cardiometabolic targets and plant-based dietary index, healthy and unhealthy, among 492 breast cancer survivors enrolled in the DEDiCa trial. Abbreviations: BMI, body mass index; LDL, low-density lipoprotein; HDL, high-density lipoprotein. ^a^ Estimated by means of logistic or multinomial regression models (when appropriate), including terms for study centre, age, years of education, physical activity, smoking status, energy intake, menopausal status, adjuvant chemotherapy and endocrine therapy. ^b^ Metabolic syndrome components were defined as (i) waist circumference ≥88 cm, (ii) systolic blood pressure ≥ 130 mmHg or diastolic blood pressure ≥ 85 mmHg or pharmacologically treated for hypertension, (iii) glycaemia ≥ 100 mmol/L or pharmacologically treated for hyperglycemia, (iv) HDL-cholesterol < 50 mg/dL or pharmacologically treated for hypercholesterolemia, and (v) triglycerides ≥ 150 mmol/L. Metabolic syndrome diagnosis is present when 3–5 components are over the target.

The associations of plant-based indices with cardiometabolic targets are reported in Figure 1 and Table 3. The OR of being obese (BMI ≥ 30 kg/m^2^) was 0.47 (95% CI: 0.29–0.77) for high versus low PDI, 0.37 (95% CI: 0.22–0.61) for hPDI and 1.38 (95% CI: 0.83–2.28) for uPDI. The OR for hypercholesterolemia (≥200 mg/dL) was 1.80 (95%CI: 1.16–2.78) for high versus low uPDI. The ORs of hypertriglyceridemia (≥150 mg/dL) and metabolic syndrome were 0.38 (95% CI: 0.20–0.71) and 0.59 (95% CI: 0.35–0.97), respectively, with higher PDI. The OR of having a large waist circumference (≥88 cm) was of borderline significance (OR = 0.64, 95% CI: 0.42–1.00) with higher hPDI. No other significant associations were observed, although the higher hPDI scores tended to be associated also with lower odds of having a BMI in the overweight ranges, i.e., 25–30 kg/m^2^ (OR = 0.63; 95% CI: 0.39–1.03), systolic blood pressure values ≥ 130 mmHg (OR = 0.54; 95% CI: 0.28–1.02) and presence of metabolic syndrome (OR = 0.64; 95% CI: 0.38–1.07).

## 4. Discussion

This cross-sectional study in Mediterranean BCS showed a lower probability of cardiometabolic risk factors outside of safety targets when adherence to a plant-based diet was higher, particularly for body weight (53–63% lower risk), serum triglycerides (62% lower risk) and metabolic syndrome (41% lower risk). Higher adherence to a healthy plant-based diet showed an additional, albeit borderline, protection from abdominal obesity (36% lower risk) while an unhealthy plant-based diet was associated with a greater risk of hypercholesterolemia (80% higher risk).

CVD remains a major cause of death in BCS [4,28], and heart failure is highly prevalent, i.e., up to 20% cumulative at 15 years postdiagnosis [47]. This is partly due to obesity and unhealthy lifestyles [48] and partly to the cardiotoxicity of oncologic treatments (i.e., anthracyclines, left-side breast radiation, trastuzumab, aromatase inhibitors). The presence of at least one CVD risk factor before or after diagnosis can further increase the risk of heart failure in BCS from 50% to 3-fold [47]. Therefore, maintaining CVD risk factors (including body weight) within target ranges before or after diagnosis is a relevant clinical target for BCS and potentially achievable with a high plant-based diet [26].

Our findings are in line with results from previous studies reporting inverse associations between plant-based diets and excess body weight [14]. In a cross-sectional study in healthy women, the risk of overweight or obesity was 65% lower in vegans, 48% lower in semi-vegetarians and 46% lower in lactovegetarians, compared to omnivores [49]. A meta-analysis including 1151 subjects demonstrated that vegetarian diets are effective in reducing body weight by an average of 2 kg in 4 months and even more in highly adherent individuals [50,51].

The Pathways study in the USA found inverse associations between higher hPDI and T2D, hypertension and dyslipidemia in BCS [26]. In line with this study, our results showed that higher hPDI significantly reduced the risk of obesity and central obesity, with a tendency to lower the risk of systolic hypertension and metabolic syndrome. Possibly our smaller sample size and the background Mediterranean diet played a role in reducing the differences of plant food intakes among our BCS, which may explain the lack of association of hPDI with cholesterolemic and glycemic targets. Our study population tended to homogenously consume some staple Mediterranean foods such as extra virgin olive oil, known to control blood lipids and glycemia [52,53], and nuts and legumes, which are two cholesterol-lowering and glycemic-lowering foods [54]. Indeed, in our study, the differences in the risk of hypercholesterolemia were seen with high uPDI because foods in this category (e.g., sugar-sweetened beverages, desserts, white bread and white rice) are not typical of the Mediterranean diet and hence not consumed by everyone. This is also in line with findings from a meta-analysis of clinical trials in non-oncologic patients, where uPDI increased serum cholesterol levels [25].

Over time, metabolic syndrome [44] increases the lifetime risk of developing T2D by 5-fold and doubles CVD risk within 10 years in non-oncologic populations [41]. In BCS, metabolic syndrome increased BC-specific mortality in a Southern Italian cohort [55]. A Chinese cross-sectional study including more than 4000 adults found that high consumption of unhealthy plant foods increased metabolic syndrome risk by 37%, abdominal obesity risk by 32%, and hypertension risk by 41% [56]. In an intervention trial, obese individuals with metabolic syndrome, substituting 60% of animal proteins with vegetable proteins such as legumes, nuts and seeds within a balanced diet (55% carbohydrates, 15% protein and 30% fat), significantly reduced body weight, waist circumference, blood pressure, glycemia and triglyceridemia compared to controls [57]. This is partly due to the lower glycemic and insulinemic potential and anti-inflammatory properties of such diets [58], contributing to reduced fat accumulation in the liver and insulin resistance related to the metabolic syndrome [59,60].

Our study showed that the cardiometabolic health benefits of plant-based diets can be achieved with a moderate decrease in the consumption of unhealthy plant foods and animal foods without their complete exclusion. A typical plant-based diet is also a characteristic of the Mediterranean diet, which has shown inverse associations with CVD and breast cancer risk and mortality [61,62,63]. The potential mechanisms explaining the inverse associations of hPDI scores with obesity, abdominal obesity, serum lipids and blood pressure in BCS may be partly explained by higher intakes of several beneficial components present in healthy plant foods such as dietary fiber, unsaturated fatty acids (MUFA and PUFA), antioxidants and micronutrients (e.g., potassium, magnesium and calcium) and lower unhealthy components such as SFA and cholesterol [12]. Vegetables, legumes, intact grains, fruits, unpeeled nuts and seeds are the main sources of dietary fiber (appetite suppressant, cholesterol and glycemic lowering), anti-inflammatory unsaturated fatty acids, antioxidant vitamins such as alpha-tocopherols and carotenoids, blood pressure regulating minerals such as potassium and magnesium and non-nutrients such as cholesterol-lowering phytosterols (especially from seeds, seed oils, olives, olive oil, nuts) and hormone-regulating phytoestrogens (especially from seeds and beans). Nuts and olives are also rich in MUFA, PUFA and polyphenols known to regulate metabolic syndrome components [64]. Beans are also a main source of anti-atherogenic amino acids [65]. These food components may promote weight loss or maintenance, reduce adiposity and potentially lower the risk of obesity through various pathways, including satiety, insulin sensitivity, reduced inflammation and gut microbiome modification [14,66]. Foods rich in dietary fiber increase satiety despite their lower caloric density and regulate fatty acid metabolism, promoting reduced adiposity [14]. Furthermore, they reduce the risk of chronic diseases and therefore may partly explain the reduced mortality [22].

The high mineral content of healthy plant foods (e.g., calcium, magnesium and potassium) is relevant to BCS who are generally at higher risk of developing hypertension and osteoporosis, sarcopenia and fatigue mainly due to oncologic treatments [67,68,69,70]. Calcium contributes to bone health, and magnesium is required for protein synthesis, for vitamin D hydroxylation, and it is essential for the regulation of muscle contractions, including those of inner arteries [71]. Potassium intake, beyond blood pressure control, beneficially affects muscle function and overall muscle health thereby preventing falls [72]. Conversely, unhealthy plant diets (highly refined grains, fruit juices, sugar-sweetened beverages, potatoes and sweets/desserts) are associated with lower dietary fiber and micronutrient content, higher glycemic index and caloric density, and unbalanced macronutrient profiles (e.g., high in sugar and saturated fat but low in protein content). This dietary pattern may lead to decreased satiety and increased hunger signals [15,73]. This could adversely affect the pathways contributing to obesity [74] and other CVD risk factors, resulting in increased chronic diseases, including breast cancer [15,16]. In our study, higher PDI and hPDI and lower uPDI showed healthier macro- and micro-nutrient profiles, i.e., higher intakes of dietary fiber, unsaturated fatty acids, antioxidant vitamins such as alpha-tocopherols and beta-carotene, minerals such as magnesium, calcium and potassium and lower intakes of sodium and saturated fats.

The strengths of our study include a large number of women diagnosed with non-metastatic breast cancer living in Italy, a country with Mediterranean dietary traditions, which allowed the study of a large range of plant-based foods. Also, the nature and type of study design, a lifestyle trial, allowed for detailed and accurate information regarding diet, body weight and serum markers of cardiovascular health. The advantage of using the three indices of plant-based diets is that they provide a quantitative measure of how closely an individual’s diet aligns with a plant-based dietary pattern. This approach addresses a significant gap in current research, which often overlooks the quality and specific types of plant foods. This method allows for studying the diet while preserving its “cultural traditions” that may include moderate consumption of animal products like fish, poultry, and fermented dairy, which have been associated with health benefits [18]. This study, however, also has limitations. The cross-sectional nature of this analysis did not allow for inferring cause–effect, but only possible associations, with limited generalizability. The tool used to collect dietary data, i.e., 7-day food records, although considered the gold standard, is subject to bias and potential distortions because it relies on self-reporting. Patients may not provide precise and valid information in estimating portion sizes, leading to potential under- or over-estimations. However, study dietitians accurately reviewed each food record with each participant during a one-hour counselling session. Residual confounding may still exist. Socioeconomic status affects purchasing power and may affect healthy food choices and the accuracy of food record reporting. However, our model included the adjustment term years of education, which is an optimal proxy of socioeconomic status in our patient population. In addition, the professional nutritional analysis software WinFood may include another source of error since the WinFood database contains a limited number of foods and recipes. Adjusting for energy intake and physical activity may partially over-adjust for variables that are mediators of dietary patterns; however, the majority of our participants were not physically active (only ~8% were very active, i.e., at least 10,000 steps per day), and there was no meaningful correlation between energy intake and physical activity. Any potential over-adjustment is therefore likely to be minor.

## 5. Conclusions

This cross-sectional analysis in BCS highlights the relevance of plant-based diets in cardiometabolic health and the importance of differentiating plant foods. Specifically, it suggests the possible protection of plant-based diets against obesity, hypertriglyceridemia and metabolic syndrome. Furthermore, a healthy plant-based diet showed possible protection from abdominal obesity and delivered the highest heart-healthy micronutrient profile and dietary fiber, while an unhealthy plant-based diet was linked to hypercholesterolemia and had a poorer micronutrient profile. These results may be relevant in oncologic clinical practice since low-quality diets, excess body weight and metabolic syndrome have been shown to increase the risk of disease recurrence and mortality among BCS.

## Figures and Tables

**Table 1:** Distribution of baseline socio-demographic characteristics, lifestyles, menopausal status, cancer stage and treatment, and cardiometabolic targets according to plant-based dietary index (PDI), healthy and unhealthy (hPDI and uPDI, respectively) among 492 breast cancer survivors enrolled in DEDiCa trial.

Variables	PDI		hPDI		uPDI	
	<52 (n = 226)	≥52 (n = 266)	<58 (n = 236)	≥58 (n = 256)	<53 (n = 213)	≥53 (n = 279)
	n (%)	n (%)	n (%)	n (%)	n (%)	n (%)
Geographical area						
Friuli Venezia Giulia	22 (9.7)	46 (17.3)	20 (8.5)	48 (18.8)	42 (19.7)	26 (9.3)
Campania	182 (80.5)	179 (67.3)	180 (76.3)	181 (70.7)	154 (72.3)	207 (74.2)
Sicily	22 (9.7)	41 (15.4)	36 (15.3)	27 (10.5)	17 (8.0)	46 (16.5)
*p*-value (χ^2^ test)	*p* < 0.01		*p* < 0.01		*p* < 0.01	
Age (years)						
<50	96 (42.5)	113 (42.5)	121 (51.3)	88 (34.4)	82 (38.5)	127 (45.5)
≥50	130 (57.5)	153 (57.5)	115 (48.7)	168 (65.6)	131 (61.5)	152 (54.5)
*p*-value (χ^2^ test)	*p* = 0.99		*p* < 0.01		*p* = 0.14	
Education (years)						
<9	85 (37.6)	69 (25.9)	83 (35.2)	71 (27.7)	49 (23.0)	105 (37.6)
9–13	77 (34.1)	100 (37.6)	82 (34.7)	95 (37.1)	78 (36.6)	99 (35.5)
≥14	64 (28.3)	97 (36.5)	71 (30.1)	90 (35.2)	86 (40.4)	75 (26.9)
*p*-value (χ^2^ test)	*p* = 0.02		*p* = 0.19		*p* < 0.01	
Physical activity (steps/day) ^a^						
<5000	120 (53.1)	111 (41.7)	114 (48.3)	117 (45.7)	81 (38.0)	150 (53.8)
≥5000	102 (45.1)	154 (57.9)	118 (50.0)	138 (53.9)	131 (61.5)	125 (44.8)
*p*-value (χ^2^ test)	*p* < 0.01		*p* = 0.53		*p* < 0.01	
Smoking status						
Never smoker	95 (42.0)	136 (51.1)	102 (43.2)	129 (50.4)	110 (51.6)	121 (43.4)
Former smoker	71 (31.4)	89 (33.5)	79 (33.5)	81 (31.6)	69 (32.4)	91 (32.6)
Current smoker	60 (26.5)	41 (15.4)	55 (23.3)	46 (18.0)	34 (16.0)	67 (24.0)
*p*-value (χ^2^ test)	*p* < 0.01		*p* = 0.20		*p* = 0.06	
Energy intake (kcal/day)						
<1400	134 (59.3)	114 (42.9)	106 (44.9)	142 (55.5)	83 (39.0)	165 (59.1)
≥1400	92 (40.7)	152 (57.1)	130 (55.1)	114 (44.5)	130 (61.0)	114 (40.9)
*p*-value (χ^2^ test)	*p* < 0.01		*p* = 0.02		*p* < 0.01	
Menopausal status						
Pre/peri-menopause	21 (9.3)	27 (10.2)	23 (9.7)	25 (9.8)	22 (10.3)	26 (9.3)
Menopause	205 (90.7)	239 (89.8)	213 (90.3)	231 (90.2)	191 (89.7)	253 (90.7)
*p*-value (χ^2^ test)	*p* = 0.46		*p* = 0.99		*p* = 0.83	
Breast cancer stages						
I	74 (32.7)	66 (24.8)	62 (26.3)	78 (30.5)	60 (28.2)	80 (28.7)
II	124 (54.9)	161 (60.5)	145 (61.4)	140 (54.7)	128 (60.1)	157 (56.3)
III	28 (12.4)	39 (14.7)	29 (12.3)	38 (14.8)	25 (11.7)	42 (15.1)
*p*-value (χ^2^ test)	*p* = 0.15		*p* = 0.31		*p* = 0.52	
Adjuvant chemotherapy						
Never	76 (33.6)	99 (37.2)	79 (33.5)	96 (37.5)	80 (37.6)	95 (34.1)
Ended before the enrollment	109 (48.2)	121 (45.5)	116 (49.2)	114 (44.5)	98 (46.0)	132 (47.3)
Ongoing	41 (18.1)	46 (17.3)	41 (17.4)	46 (18.0)	35 (16.4)	52 (18.6)
*p*-value (χ^2^ test)	*p* = 0.71		*p* = 0.56		*p* = 0.67	
Endocrine therapy ^b^						
Never	73 (32.3)	83 (31.2)	81 (34.3)	75 (29.3)	70 (32.9)	86 (30.8)
Ongoing	150 (66.4)	182 (68.4)	154 (65.3)	178 (69.5)	142 (66.7)	190 (68.1)
*p*-value (χ^2^ test)	*p* = 0.62		*p* = 0.30		*p* = 0.73	
BMI (kg/m^2^) ^c^						
<25	71 (31.4)	118 (44.4)	74 (31.4)	115 (44.9)	97 (45.5)	92 (33.0)
25–<30	61 (27.0)	88 (33.1)	73 (30.9)	76 (29.7)	60 (28.2)	89 (31.9)
≥30	94 (41.6)	60 (22.6)	89 (37.7)	65 (25.4)	56 (26.3)	98 (35.1)
*p*-value (χ^2^ test)	*p* < 0.01		*p* < 0.01		*p* = 0.01	
Waist circumference (cm)						
<88	63 (27.9)	90 (33.8)	64 (27.1)	89 (34.8)	75 (35.2)	78 (28.0)
≥88	163 (72.1)	176 (66.2)	172 (72.9)	167 (65.2)	138 (64.8)	201 (72.0)
*p*-value (χ^2^ test)	*p* = 0.19		*p* = 0.08		*p* = 0.10	
Systolic blood pressure (mmHg) ^a^						
<130	147 (65.0)	203 (76.3)	163 (69.1)	187 (73.0)	159 (74.6)	191 (68.5)
≥130	71 (31.4)	61 (22.9)	68 (28.8)	64 (25.0)	52 (24.4)	80 (28.7)
*p*-value (χ^2^ test)	*p* = 0.03		*p* = 0.39		*p* = 0.28	
Diastolic blood pressure (mmHg) ^a^						
<85	153 (67.7)	207 (77.8)	163 (69.1)	197 (77.0)	165 (77.5)	195 (69.9)
≥85	65 (28.8)	57 (21.4)	68 (28.8)	54 (21.1)	46 (21.6)	76 (27.2)
*p*-value (χ^2^ test)	*p* = 0.05		*p* = 0.06		*p* = 0.14	
Glycemia (mmol/L)						
<100	173 (76.5)	218 (82.0)	192 (81.4)	199 (77.7)	167 (78.4)	224 (80.3)
≥100	53 (23.5)	48 (18.0)	44 (18.6)	57 (22.3)	46 (21.6)	55 (19.7)
*p*-value (χ^2^ test)	*p* = 0.17		*p* = 0.38		*p* = 0.69	
Glycated hemoglobin (%)						
<6	195 (86.3)	240 (90.2)	209 (88.6)	226 (88.3)	193 (90.6)	242 (86.7)
≥6	31 (13.7)	26 (9.8)	27 (11.4)	30 (11.7)	20 (9.4)	37 (13.3)
*p*-value (χ^2^ test)	*p* = 0.22		*p* = 0.99		*p* = 0.24	
Total cholesterol (mg/dL)						
<200	132 (58.4)	149 (56.0)	126 (53.4)	155 (60.5)	135 (63.4)	146 (52.3)
≥200	94 (41.6)	117 (44.0)	110 (46.6)	101 (39.5)	78 (36.6)	133 (47.7)
*p*-value (χ^2^ test)	*p* = 0.66		*p* = 0.13		*p* = 0.02	
LDL-cholesterol (mg/dL) ^a^						
<116	100 (44.2)	107 (40.2)	97 (41.1)	110 (43.0)	100 (46.9)	107 (38.4)
≥116	125 (55.3)	159 (59.8)	138 (58.5)	146 (57.0)	113 (53.1)	171 (61.3)
*p*-value (χ^2^ test)	*p* = 0.39		*p* = 0.77		*p* = 0.07	
HDL-cholesterol (mg/dL) ^a^						
<50	78 (34.5)	76 (28.6)	74 (31.4)	80 (31.2)	64 (30.0)	90 (32.3)
≥50	147 (65.0)	190 (71.4)	161 (68.2)	176 (68.8)	149 (70.0)	188 (67.4)
*p*-value (χ^2^ test)	*p* = 0.18		*p* = 0.99		*p* = 0.65	
Triglycerides (mmol/L)						
<150	180 (79.6)	237 (89.1)	199 (84.3)	218 (85.2)	184 (86.4)	233 (83.5)
≥150	46 (20.4)	29 (10.9)	37 (15.7)	38 (14.8)	29 (13.6)	46 (16.5)
*p*-value (χ^2^ test)	*p* < 0.01		*p* = 0.90		*p* = 0.45	
Metabolic syndrome (components) ^b^						
0–1	77 (34.1)	124 (46.6)	91 (38.6)	110 (43.0)	95 (44.6)	106 (38.0)
2	63 (27.9)	72 (27.1)	66 (28.0)	69 (27.0)	51 (23.9)	84 (30.1)
3–5	86 (38.1)	70 (26.3)	79 (33.5)	77 (30.1)	67 (31.5)	89 (31.9)
*p*-value (χ^2^ test)	*p* < 0.01		*p* = 0.58		*p* = 0.23	

Abbreviations: BMI, body mass index; LDL, low-density lipoprotein; HDL, high-density lipoprotein. ^a^ The sum does not add up to the total because of missing values (n = 5 for physical activity; n = 10 for blood pressure; n = 1 for LDL- and n = 1 for HDL-cholesterol). ^b^ Metabolic syndrome components were defined as (i) waist circumference ≥ 88 cm, (ii) systolic blood pressure ≥ 130 mmHg or diastolic blood pressure ≥ 85 mmHg or pharmacologically treated for hypertension, (iii) glycaemia ≥ 100 mmol/L or pharmacologically treated for hyperglycemia, (iv) HDL-cholesterol < 50 mg/dL or pharmacologically treated for hypercholesterolemia, and (v) triglycerides ≥ 150 mmol/L; ^c^ BMI < 25 included also three subjects with BMI < 18.5 kg/m^2^.

**Table 2:** Distribution of baseline food groups, plant-based dietary index (PDI), healthy and unhealthy (hPDI and uPDI, respectively) among 492 breast cancer survivors enrolled in DEDiCa trial.

Food Groups	Median (Q1–Q2)	
Healthy plant food group (g/day)		
Wholegrains (breakfast cereals, other cooked breakfast cereals, cooked oatmeal, dark bread, brown rice, other grains, bran, wheat germ, popcorn, pasta made with whole grains and with durum wheat semolina flour)	60.0	(41.4–82.9)
Fruit	150.0	(81.0–238.5)
Vegetables	163.6	(102.9–231.6)
Nuts (nuts and peanut butter)	1.1	(0.0–6.0)
Legumes (all legumes, including tofu and soybeans)	14.3	(4.3–28.6)
Vegetables oils	17.1	(12.1–22.9)
Tea and coffee	85.7	(47.3–148.6)
Less healthy plant food group (g/day)		
Fruit juices	0.0	(0.0–17.9)
Sugar-sweetened beverages (colas with caffeine and sugar, colas without caffeine but with sugar, other carbonated beverages with sugar, noncarbonated fruit drinks with sugar)	0.0	(0.0–0.0)
Refined grains (breakfast cereals, white bread, English muffins or bagel or rolls, muffins or biscuits, white rice, pancakes or waffles, crackers)	60.7	(40.0–89.7)
Potatoes	14.3	(0.0–28.6)
Sweets/desserts [chocolates, candy bars, candies without chocolate, cookies (home-made and commercial), brownies, cakes (home-made and commercial), sweet-rolls (home-made and commercial), pies (home-made and commercial), jams or jellies or preserves or syrup or honey]	47.3	(29.0–71.2)
Animal food group (g/day)		
Animal fat (butter added to food, butter or lard used for cooking)	0.0	(0.0–0.0)
Dairy	90.8	(32.9–180.0)
Eggs	8.6	(0.0–17.1)
Fish/seafood	41.4	(20.0–65.7)
Meat	59.7	(38.6–85.2)
Miscellaneous animal-based foods (pizza, chowder or creamy soups, mayonnaise or other creamy salad dressings)	28.6	(0.0–50.0)
Plant-based dietary index, PDI (score)	52	(48–57)
Healthy plant-based dietary index, hPDI (score)	58	(53–62)
Unhealthy plant-based dietary index, uPDI (score)	53	(49–58)

**Table 3:** Odd ratios (ORs) and 95% confidence intervals (CIs) of having cardiometabolic targets outside healthy ranges according to higher vs. lower overall plant-based dietary index (PDI), healthy and unhealthy (hPDI and uPDI, respectively) in 492 breast cancer survivors enrolled in DEDiCa trial.

Cardiometabolic Targets	PDI	hPDI	uPDI
	OR (95% CI) ^a^	OR (95% CI) ^a^	OR (95% CI) ^a^
BMI (median: 26.6 kg/m^2^; IQR: 23.6–31.2) ^c^			
<25	Ref	Ref	Ref
25–<30	1.04 (0.64–1.68)	0.63 (0.39–1.03)	1.31 (0.81–2.11)
≥30	0.47 (0.29–0.77)	0.37 (0.22–0.61)	1.38 (0.83–2.28)
Waist circumference (median: 94.8 cm; IQR: 86.0–105.0) ^c^			
<88	Ref	Ref	Ref
≥88	0.94 (0.61–1.45)	0.64 (0.42–1.00)	1.25 (0.81–1.93)
Systolic blood pressure (median: 120.0 mmHg; IQR: 110.0–71.0) ^c^			
<130	Ref	Ref	Ref
≥130	0.54 (0.29–1.02)	0.54 (0.28–1.02)	1.17 (0.62–2.21)
Diastolic blood pressure (median: 77.0 mmHg; IQR: 71.0–85.0) ^c^			
<85	Ref	Ref	Ref
≥85	0.62 (0.35–1.09)	0.65 (0.37–1.15)	0.91 (0.51–1.61)
Glycemia (median: 88.0 mmol/L; IQR: 81.0–96.8) ^c^			
<100	Ref	Ref	Ref
≥100	0.77 (0.47–1.26)	1.09 (0.67–1.80)	0.77 (0.46–1.28)
Glycated hemoglobin (median: 5.3%; IQR: 4.9–5.7) ^c^			
<6	Ref	Ref	Ref
≥6	0.88 (0.48–1.62)	1.03 (0.56–1.90)	0.97 (0.51–1.83)
Total cholesterol (median: 192.0 mg/dL; IQR: 171.0–220.0) ^c^			
<200	Ref	Ref	Ref
≥200	1.08 (0.71–1.64)	0.68 (0.45–1.03)	1.80 (1.16–2.78)
LDL-cholesterol (median: 121.0 mg/dL; IQR: 101.0–145.0) ^c^			
<116	Ref	Ref	Ref
≥116	1.34 (0.88–2.05)	0.80 (0.53–1.22)	1.46 (0.95–2.25)
HDL-cholesterol (median: 56.0 mg/dL: IQR: 47.0–66.0) ^c^			
<50	0.77 (0.49–1.21)	0.92 (0.59–1.45)	0.92 (0.58–1.47)
≥50	Ref	Ref	Ref
Triglycerides (median: 94.0 mmol/L; IQR: 70.0–126.0) ^c^			
<150	Ref	Ref	Ref
≥150	0.38 (0.20–0.71)	0.72 (0.39–1.32)	1.06 (0.56–2.00)
Metabolic syndrome (components) ^b^			
0–1	Ref	Ref	Ref
2	0.79 (0.49–1.28)	0.80 (0.50–1.31)	1.31 (0.80–2.14)
3–5	0.59 (0.35–0.97)	0.64 (0.38–1.07)	0.87 (0.52–1.45)

Abbreviations: BMI, body mass index; HDL, high-density lipoprotein; LDL, low-density lipoprotein. ^a^ Estimated by means of logistic or multinomial regression models (when appropriate), including terms for study centre, age, years of education, physical activity, smoking status, energy intake, menopausal status, adjuvant chemotherapy and endocrine therapy. ^b^ Metabolic syndrome components were defined as (i) waist circumference ≥ 88 cm, (ii) systolic blood pressure ≥ 130 mmHg or diastolic blood pressure ≥ 85 mmHg or pharmacologically treated for hypertension, (iii) glycaemia ≥ 100 mmol/L or pharmacologically treated for hyperglycemia, (iv) HDL-cholesterol < 50 mg/dL or pharmacologically treated for hypercholesterolemia, and (v) triglycerides ≥ 150 mmol/L. ^c^ Values of median and interquartile ranges of cardiomeabolic risk factors were indicated in brackets.

## Data Availability

The data presented in this study are available on request from the last author.

## Supplementary Materials

The following supporting information can be downloaded at https://www.mdpi.com/article/10.3390/nu17233782/s1: Figure S1: DAG showing the covariates used to adjust the estimates of BMI related to plant-based diet indices. Figure S2: Mean selected diet quality indicators according to high and low plant-based diet indices (N = 492); Table S1: STROBE Statement—Checklist of items included in reports of cross-sectional studies; Table S2: Distribution of baseline dietary variables by median scores of overall plant-based dietary index (PDI), healthy and unhealthy (hPDI and uPDI, respectively) in 492 breast cancer survivors enrolled in DEDiCa trial.

## Abbreviations

The following abbreviations are used in this manuscript:

AIFA	Italian Drug Agency
BCSs	Breast Cancer Survivors
BDA	Banca Dati di composizione degli Alimenti per studi epidemiologici in Italia
BMI	Body Mass Index
CI	Confidence Interval
CREA	Consiglio per la Ricerca in Agricoltura e l’Analisi dell’Economia Agraria
CVD	Cardiovascular Disease
DAG	Directed Acyclic Graph
EAS	European Atherosclerosis Society
EDTA	Ethylene Diamine Tetra Acetic Acid
ESC	European Society of Cardiology
FAO	Food and Agriculture Organization
GI	Glycemic Index
HbA1c	Hemoglobin A1c
HDL-C	High Density Lipoprotein—Cholesterol
hPDI	Healthy Plant-based Dietary Index
IQR	Interquartile Range
ISO	International Organization for Standardization
LDL-C	Low Density Lipoprotein—Cholesterol
MEDAS	Mediterranean Diet Adherence Screener
MUFA	Mono-Unsaturated Fatty Acid
NCEP	National Cholesterol Education Program
OR	Odds Ratio
PDI	Plant-based Dietary Index
PUFA	Poly-Unsaturated Fatty Acid
SFA	Saturated Fatty Acid
T2D	Type 2 Diabetes
uPDI	Unhealthy Plant-based Dietary Index
WHO	World Health Organization
