# Supplementary material for: Healthful and Unhealthful Plant-Based Diets and Their Association with Cardiometabolic Targets in Women Diagnosed with Breast Cancer: A Cross-Sectional Analysis of a Lifestyle Trial
Source: Nutrients. 2025 Dec 2;17(23):3782. doi: 10.3390/nu17233782 (PMC12694399; doi:10.3390/nu17233782)
Supplement: Supplementary file 1 [file nutrients-17-03782-s001.zip › nutrients-3987346-supplementary.pdf]

Table S1. STROBE Statement—Checklist of items included in reports of cross-sectional studies

	Item No	Recommendation	Reported on page and tables/figures No.
**Title and abstract**	1	(*a*) Indicate the study's design with a commonly used term in the title or the abstract	1
		(*b*) Provide in the abstract an informative and balanced summary of what was done and what was found	2
**Introduction**			
Background/rationale	2	Explain the scientific background and rationale for the investigation being reported	2-3
Objectives	3	State specific objectives, including any prespecified hypotheses	2-3
**Methods**			
Study design	4	Present key elements of study design early in the paper	3
Setting	5	Describe the setting, locations, and relevant dates, including periods of recruitment, exposure, follow-up, and data collection	3-5
Participants	6	(*a*) Give the eligibility criteria, and the sources and methods of selection of participants	3-4
Variables	7	Clearly define all outcomes, exposures, predictors, potential confounders, and effect modifiers. Give diagnostic criteria, if applicable	4-5
Data sources/ measurement	8*	For each variable of interest, give sources of data and details of methods of assessment (measurement). Describe comparability of assessment methods if there is more than one group	3-5
Bias	9	Describe any efforts to address potential sources of bias	3
Study size	10	Explain how the study size was arrived at	3
Quantitative variables	11	Explain how quantitative variables were handled in the analyses. If applicable, describe which groupings were chosen and why	3-5
Statistical methods	12	(*a*) Describe all statistical methods, including those used to control for confounding	6
		(*b*) Describe any methods used to examine subgroups and interactions	6
		(*c*) Explain how missing data were addressed	6
		(*d*) If applicable, describe analytical methods taking account of sampling strategy	n/a
		(*e*) Describe any sensitivity analyses	n/a
**Results**			

Participants	13*	(a) Report numbers of individuals at each stage of study—eg numbers potentially eligible, examined for eligibility, confirmed eligible, included in the study, completing follow-up, and analysed	7 and Table 1
		(b) Give reasons for non-participation at each stage	n/a
		(c) Consider use of a flow diagram	n/a
Descriptive data	14*	(a) Give characteristics of study participants (eg demographic, clinical, social) and information on exposures and potential confounders	7-10, Table 1, 2, S2 and Figure S1
		(b) Indicate number of participants with missing data for each variable of interest	6, 9
Outcome data	15*	Report numbers of outcome events or summary measures	Table 1, 2, S2 and Figure S1
Main results	16	(*a*) Give unadjusted estimates and, if applicable, confounder-adjusted estimates and their precision (eg, 95% confidence interval). Make clear which confounders were adjusted for and why they were included	11-13, Figure 1 and Table 3
		(*b*) Report category boundaries when continuous variables were categorized	Table 2, S2
		(*c*) If relevant, consider translating estimates of relative risk into absolute risk for a meaningful time period	n/a
Other analyses	17	Report other analyses done—eg analyses of subgroups and interactions, and sensitivity analyses	11, Table S2 and Figure S1
**Discussion**			
Key results	18	Summarise key results with reference to study objectives	13
Limitations	19	Discuss limitations of the study, taking into account sources of potential bias or imprecision. Discuss both direction and magnitude of any potential bias	13-15
Interpretation	20	Give a cautious overall interpretation of results considering objectives, limitations, multiplicity of analyses, results from similar studies, and other relevant evidence	13-15
Generalisability	21	Discuss the generalisability (external validity) of the study results	15-16
**Other information**			
Funding	22	Give the source of funding and the role of the funders for the present study and, if applicable, for the original study on which the present article is based	16

Abbreviations: n/a=not applicable

*Give information separately for exposed and unexposed groups.

Note: Information on the STROBE Initiative is available at www.strobe-statement.org.

Table S2. Distribution of baseline dietary variables by median scores of overall plant-based dietary index (PDI), healthy and unhealthy (hPDI and uPDI, respectively) in 492 breast cancer survivors enrolled in DEDiCa trial.

**Dietary variables**	**PDI**			**hPDI**			**uPDI**		
	**<52**	**≥52**	***p*[a]**	**<58**	**≥58**	***p*[a]**	**<53**	**≥53**	***p*[a]**
	mean ± SD (%E)	mean ± SD (%E)		mean ± SD (%E)	mean ± SD (%E)		mean ± SD (%E)	mean ± SD (%E)	
**Energy intake** *Kcal*	1344.0 ± 310.7	1491.9 ± 336.6	**<0.001**	1472.8 ± 345.3	1378.9 ± 315.1	**0.002**	1514.6 ± 321.7	1354.7 ± 325.2	**<0.001**
**Carbohydrates** *g/1000Kcal*	126.1 ± 18.1 (47)	129.8 ± 16.9 (49)	**0.02**	129.2 ± 16.2 (49)	127.0 ± 18.7 (48)	**0.01**	123.7 ± 17.3 (46)	131.5 ± 17.0 (49)	**<0.001**
**Proteins** *g/1000Kcal*	43.4 ± 7.6 (17)	40.0 ± 7.1 (16)	**0.001**	41.2 ± 7.2 (17)	41.9 ± 7.8 (17)	0.3	43.2 ± 7.6 (17)	40.3 ± 7.2 (16)	**<0.001**
**Fats** *g/1000 Kcal*	38.2 ± 6.9 (34)	38.0 ± 6.6 (34)	0.64	37.8 ± 6.0 (34)	38.3 ± 7.3 (35)	0.4	39.2 ± 6.5 (35)	37.2 ± 6.8 (34)	**0.001**
**SFA** *g/1000 Kcal*	11.7 ± 2.5 (11)	10.6 ± 2.8 (9.6)	**<0.001**	12.1 ± 2.6 (11)	10.2 ± 2.6 (9)	**<0.001**	10.8 ± 2.6 (10)	11.4 ± 2.8 (10)	**0.02**
**MUFA** *g/1000 Kcal*	17.3 ± 4.9 (16)	17.5 ± 4.3 (16)	0.65	16.2 ± 3.7 (15)	18.5 ± 5.1 (17)	0.7	18.5 ± 4.6 (17)	16.5 ± 4.5 (15)	**<0.001**
**PUFA** *g/1000 Kcal*	5.0 ± 1.3 (4)	5.4 ± 1.5 (5)	**0.002**	5.0 ± 1.2 (5)	5.4 ± 1.5 (5)	**0.002**	5.5 ± 1.4 (5)	5.0 ± 1.4 (4)	**<0.001**
**Dietary cholesterol** *mg/1000 Kcal*	132.1 ± 45.1	106.1 ± 36.6	**<0.001**	124.4 ± 40.2	112.2 ± 44.2	**0.001**	125.3 ± 44.0	112.5 ± 40.9	**0.001**
**Dietary fiber** g/*1000 Kcal*	11.5 ± 3.7	13.8 ± 4.0	**<0.001**	10.6 ± 2.8	14.6 ± 4.0	**<0.001**	14.5 ± 3.9	11.4 ± 3.5	**<0.001**
**Glycemic load** /1000 Kcal	71.7 ± 11.9	72.9 ± 12.3	0.30	74.6 ± 10.9	70.2 ± 12.9	**<0.001**	68.1 ± 11.6	75.6 ± 11.6	**<0.001**
**Glycemic index**	56.8 ± 4.0	56.0 ± 4.3	**0.03**	57.7 ± 3.7	55.1 ± 4.2	**<0.001**	54.9 ± 3.9	57.4 ± 4.1	**<0.001**
**Mediterranean diet adherence[b]**	7.5 ± 1.9	8.4 ± 1.9	**<0.001**	7.3 ± 1.9	8.6 ± 1.8	**<0.001**	8.7 ± 1.8	7.4 ± 1.9	**<0.001**
**Sodium, Na** *mg/1000 Kcal*	1033.3 ± 326.2	834.4 ± 269.1	**<0.001**	1017.7 ± 301.2	841.0 ± 299.0	**<0.001**	894.4 ± 295.9	949.7 ± 323.1	0.05
**Potassium, K** *mg/1000 Kcal*	1497.6 ± 390.5	1669.1 ± 423.8	**<0.001**	1427.2 ± 307.6	1740.7 ± 448.1	**<0.001**	1757.6 ± 425.8	1462.6 ± 362.6	**<0.001**
**Magnesium, Mg** *mg/1000 Kcal*	97.7 ± 27.8	115.4 ± 37.6	**<0.001**	92.3 ± 25.1	121.0 ± 36.4	**<0.001**	119.6 ± 35.0	97.8 ± 31.1	**<0.001**
**Calcium, Ca** *mg/1000 kcal*	328.9 ± 107.4	315.6 ± 105.3	0.17	315.8 ± 104.7	327.2 ± 107.8	0.2	347.2 ± 103.2	302.3 ± 104.8	**<0.001**

**Iron, Fe** *mg/1000 kcal*	5.9 ± 1.7	6.6 ± 1.9	**<0.001**	5.6 ± 1.4	6.9 ± 2.0	**<0.001**	7.0 ± 1.9	5.7 ± 1.6	**<0.001**
**Folic acid, vitamin B9** *mcg/1000 kcal*	164.0 ± 70.2	186.0 ± 76.6	**0.001**	145.8 ± 52.9	203.7 ± 80.6	**0.001**	200.6 ± 76.5	157.1 ± 67.1	**<0.001**
**α-tocopherol** *mg/1000 Kcal*	0.5 ± 0.3	0.6 ± 0.3	**<0.001**	0.4 ± 0.2	0.6 ± 0.4	**<0.001**	0.6 ± 0.3	0.5 ± 0.3	**<0.001**
**β-carotene** *mg/1000 Kcal*	872.2 ± 648.3	1199.9 ± 1081.9	**<0.001**	825.9 ± 546.3	1255.4 ± 1129.0	**<0.001**	1300.3 ± 1184.8	857.8 ± 589.5	**<0.001**

Abbreviations: SD, standard deviation; %E, percent of total daily energy intake; SFA, saturated fatty acids; MUFA, monounsaturated fatty acids; PUFA, polyunsaturated fatty acids; vit., vitamin.

[a] *p-value from t-test for differences between over and under the median value of PDIs;* [b] *assessed by the* Mediterranean diet adherence screener *MEDAS.*

Figure S1. DAG showing the covariates used to adjust the estimates of BMI related to plant-based diet indices.

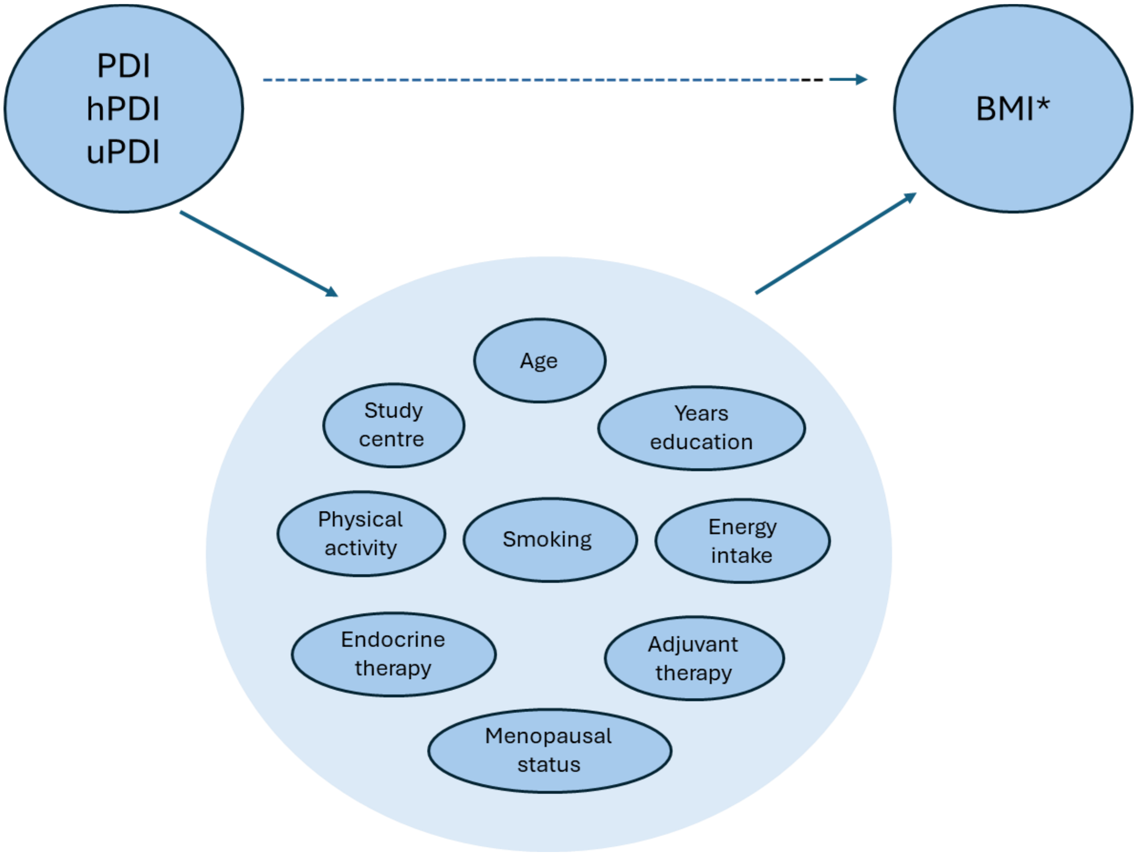

*Same DAG applies for all other dependent variables, i.e. waist circumference, systolic and diastolic blood pressure, fasting blood glucose, hemoglobin A1c, LDL- and HDL-cholesterol, triglycerides and metabolic syndrome.

Figure S2. Mean selected diet quality indicators according to high and low plant-based diet indices (N=492)

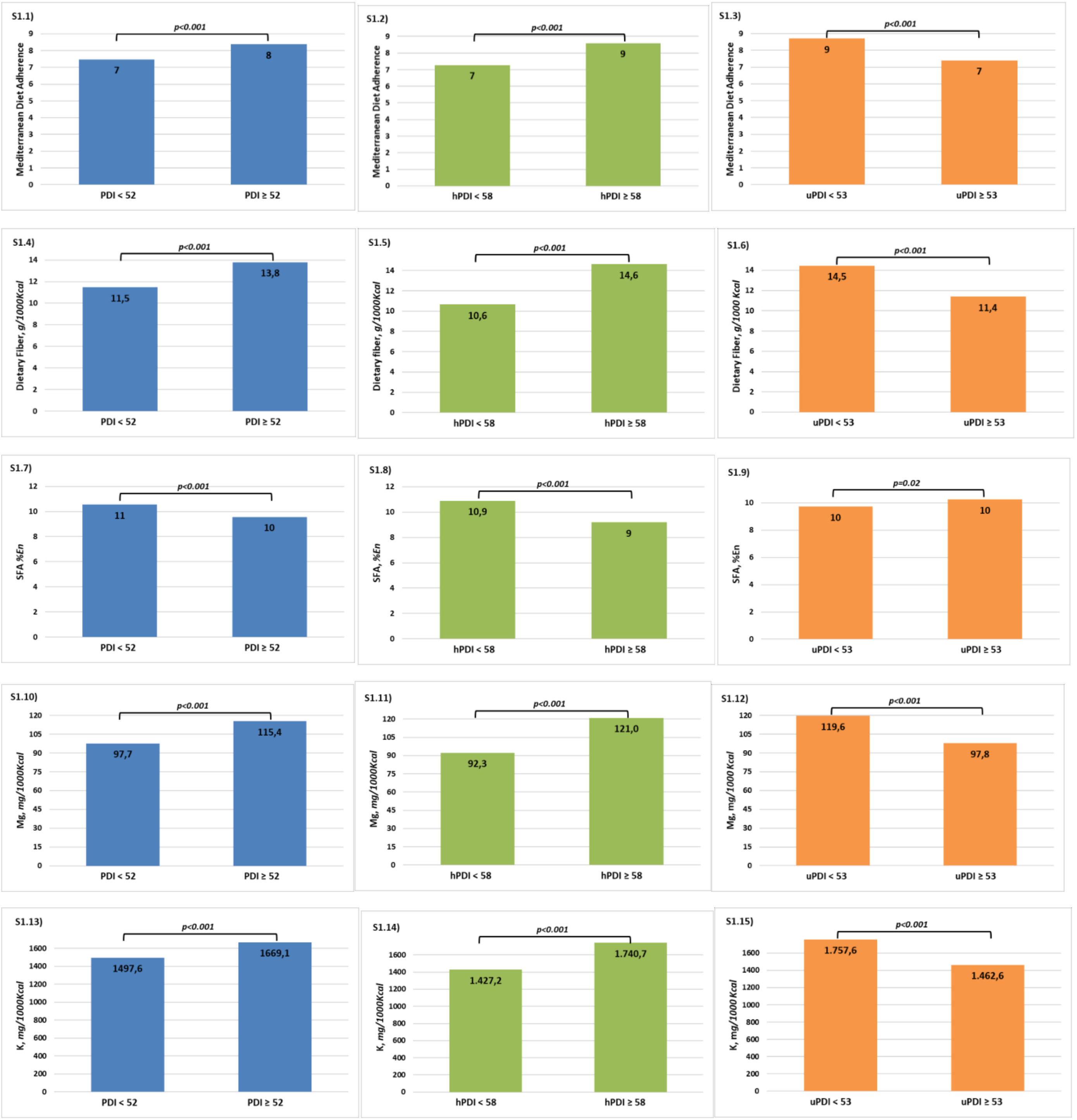

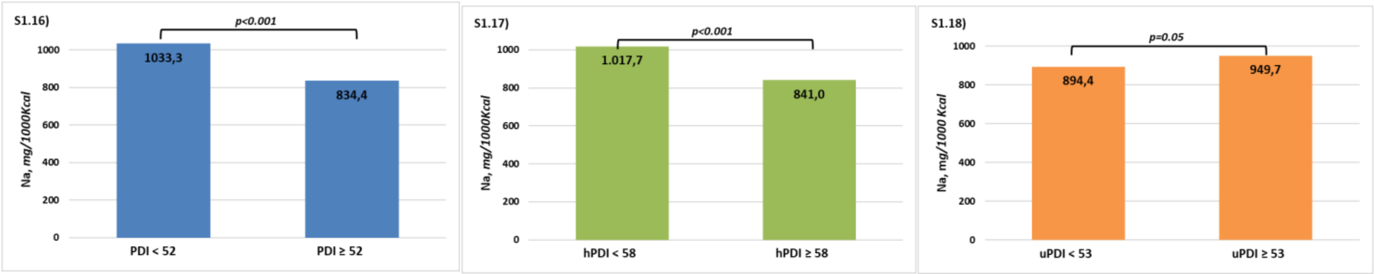

Mediterranean diet adherence calculated by Mediterranean diet adherence screener (MEDAS), is a 14-items validated score with a maximum of 14 and minimum of 0, indicating higher and lower adherence to the Mediterranean diet, respectively.

Abbreviations: PDI, plant-based dietary index; hPDI, healthy plant-based dietary index; uPDI, unhealthy plant-based dietary index; %E, percent on energy intake; SFA, saturated fatty acids; Mg, magnesium; K, potassium; Na, sodium. *p-value from t-test for differences between values of PDIs over and under the median.*
